# Supplementary material for: Complex‐centric proteome profiling by SEC‐SWATH‐MS
Source: Mol Syst Biol. 2019 Jan 14;15(1):e8438. doi: 10.15252/msb.20188438 (PMC6346213; doi:10.15252/msb.20188438)
Supplement: Supplementary file 8 — Dataset EV7 [file MSB-15-e8438-s008.zip › feature_plots_string/Q5T653.pdf]

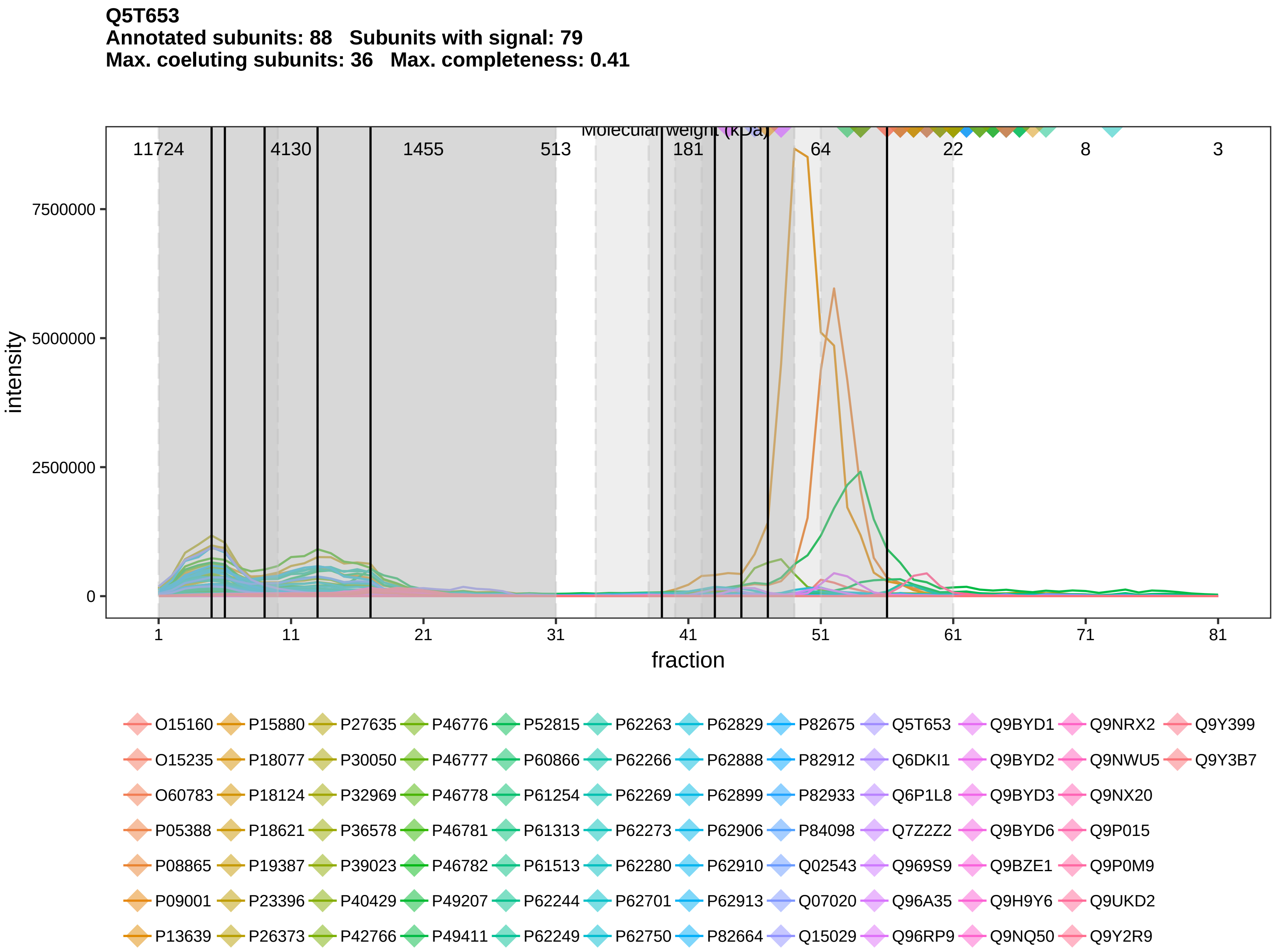
Q5T653
Annotated subunits: 88   Subunits with signal: 79
Max. coeluting subunits: 36   Max. completeness: 0.41
Molecular weight (kDa)
11724
4130
1455
513
181
64
22
8
3
intensity
7500000
5000000
2500000
0
fraction
1
11
21
31
41
51
61
71
81
O15160
O15235
O60783
P05388
P08865
P09001
P13639
P15880
P18077
P18124
P18621
P19387
P23396
P26373
P27635
P30050
P32969
P36578
P39023
P40429
P42766
P46776
P46777
P46778
P46781
P46782
P49207
P49411
P52815
P60866
P61254
P61313
P61513
P62244
P62249
P62263
P62266
P62269
P62273
P62280
P62701
P62750
P62829
P62888
P62899
P62906
P62910
P62913
P82664
P82675
P82912
P82933
P84098
Q02543
Q07020
Q15029
Q5T653
Q6DKI1
Q6P1L8
Q7Z2Z2
Q969S9
Q96A35
Q96RP9
Q9BYD1
Q9BYD2
Q9BYD3
Q9BYD6
Q9BZE1
Q9H9Y6
Q9NQ50
Q9NRX2
Q9NWU5
Q9NX20
Q9P015
Q9P0M9
Q9UKD2
Q9Y2R9
Q9Y399
Q9Y3B7
